# Advancing Computerized Cognitive Training for MCI and Alzheimer's Disease in a Pandemic and Post-pandemic World

**DOI:** 10.3389/fpsyt.2020.557571

**Published:** 2020-11-25

**Authors:** Kaylee A. Bodner, Terry E. Goldberg, D. P. Devanand, P. Murali Doraiswamy

**Affiliations:** ^1^Neurocognitive Disorders Program, Departments of Psychiatry and Medicine, Duke University School of Medicine, Durham, NC, United States; ^2^Division of Geriatric Psychiatry, New York State Psychiatric Institute, New York, NY, United States; ^3^Department of Psychiatry, Columbia University Irving Medical Center, New York, NY, United States

**Keywords:** aging, dementia, cognitive reserve, COVID long hauler, digital therapeutic

## Introduction

Worldwide, some 40 million adults have Alzheimer's disease (AD) (1) and several hundred million may be at elevated risk for AD by virtue of mild cognitive impairment (MCI) and/or silent buildup of cortical AD pathology. There are no pharmacological treatments with more than minimal efficacy for mild AD, and prevention strategies are not established.

The COVID-19 pandemic has transformed mobile health applications and telemedicine from nice to have tools into essential healthcare infrastructure (2–13). We anticipate that this need will be particularly great for the elderly who, due to their greater risk for infection, may avoid medical facilities or be required to self-isolate. These are also the very groups at highest risk for cognitive decline. Further, emerging data suggests COVID-19 may itself be linked with longer-term neurological consequences, including cognitive decline (5).

Definitive data on the utility of cognitive/mental wellness tools during the pandemic awaits the results of ongoing clinical trials (6–10), but there is accumulating preliminary evidence (11). For example, during the COVID-19 pandemic, chatbots employed by hospitals and government agencies fielded millions of queries from concerned patients (3). Digital tools also were deployed to provide psychological self-help to people isolated at home or in retirement centers and nursing homes (2–4). A survey of 1,000 adults done in March 2020 (12) found that 82% were concerned about leaving their home, 78% are avoiding doctor visits unrelated to COVID and 80% would prefer to receive a remote virtual health consultation if given the opportunity. A recent survey of elderly MCI subjects during the pandemic (13) demonstrated potential for cognitive stimulation *via* assistive technology but also found that those living alone had the greatest negative mental effects.

## Computerized Cognitive Training

**Figure 1:**
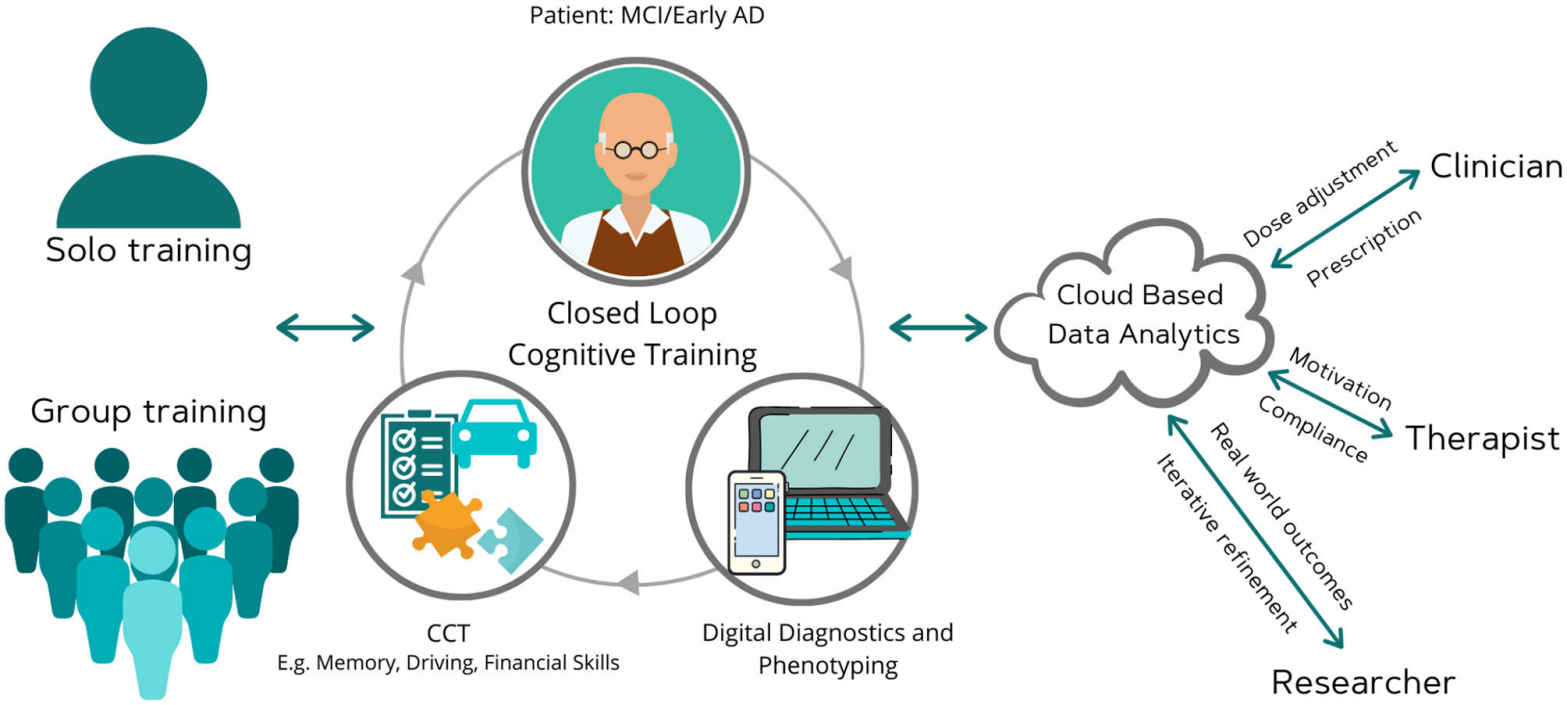
Moving computerized cognitive training toward a digital brain therapeutic. This scheme envisions a remote system that integrates digital diagnostics and therapeutics, and is cleared by regulators on the basis of well-controlled efficacy trials or through a pre-certification program. Such a digital therapeutic would allow for remote compliance monitoring and dose adjustment by clinicians. It would also allow for iterative software refinement by developer and real world evidence collection by researchers. Clinician, researcher, and therapist are depicted as distinct individuals for ease of showing the capabilities. The software would permit these roles to overlap, i.e., the clinician may also serve as a therapist and researcher.

Computerized cognitive training (CCT) is one such application of digital health in which individuals can access gamified, engaging, cognitive exercises from their own computers or mobile devices anytime anywhere (14–24). These exercises can be targeted to improve overall cognition or specific domains (such as learning and memory, attention, speed, executive functioning), as well as daily living skills such as financial knowledge or driving performance (14–24). They can potentially be adjusted based on response *via* self-administered cognitive tests, and adherence supervised remotely, as needed, by a physician or psychologist (Figure 1).

## Clinical Trials of CCT in Aging and MCI

While scientific opinion regarding CCT has in the past been divided (15–18), there is now a growing body of evidence (pre-COVID) from pre-clinical and observational research [reviewed in (14, 20–24)] as well as data from large RCTs and meta-analyses to show that certain cognitive training regimens can improve cognitive and functional abilities in older adults (17). For example, the NIH-funded, Advanced Cognitive Training for Independent and Vital Elderly (ACTIVE) Trial of 2,832 older people, assigned people to 3 forms of training—memory, reasoning and speed—vs. a control. The memory group showed no benefits. But 5 years after initial training, the reasoning group self-reported fewer daily-living problems, whereas speed-of-processing training resulted in fewer at-fault automobile accidents and a smaller decline in health-related quality of life (21). Further, at 10-year follow up, those on the computerized speed training arm had a 29% reduction in incident dementia rates (22). Likewise, while an initial online study by Owen et al. (18) did not find benefits of CCT in younger adults, a subsequent study of 2,912 older adults by the same group reported that CCT had benefits on both cognition and daily activities (19). A meta-analyses of 52 studies comprising 4,885 cognitively healthy older adults, noted small to moderate beneficial effect sizes for CCT in comparison to control groups in the domains of verbal memory, non-verbal memory, working memory, processing speed, and visuospatial skills (23). This study also found that group-based training was more efficacious than home-based training—suggesting that future home based CCT may need to be augmented with greater remote supervision and interactions *via* social media (23). A meta-analysis of 18 studies of CCT for MCI (*N* = 690) found small to moderate improvements in global cognition, memory and working memory (20). The largest effect size was on working memory. Whether these improvements result in long-term transfer to clinically meaningful benefits and lowered rates of progression to dementia is not known and require further study (20).

There is also evidence that the effectiveness of CCT in subjects at risk for AD could be improved by supplementing cognitive training with other tools such as physical exercise, diet, vascular risk reduction, neuromodulation or pharmacotherapy. For example, Lenze et al. (25) reported that the addition of a serotonin modulator/stimulator drug, vortioxetine, could improve the efficacy of CCT in MCI. Two studies that examined the effects of combining physical and cognitive training in MCI reported mixed results (24, 26). Singh et al. (24), using a 2 × 2 design, found that CCT improved memory in MCI at 6 months but did not augment the effects of exercise. In contrast, the 40-week population study by Shimada et al. (26) of 945 MCI subjects reported that combined CCT and physical exercise improved memory and non-memory domains, and reduced medial temporal lobe atrophy in amnestic MCI (26). Lastly, the 2-year FINGER randomized controlled trial of 1,260 older adults showed that a multi-domain lifestyle intervention, comprising CCT as one of the components, slowed cognitive decline (27). While the benefits seen in multi-domain intervention studies cannot be attributed solely to CCT, these data, together with data from monotherapy RCTs, support further development of CCT for cognitive rehabilitation of MCI and early AD.

## Advancing CCT as a Digital Brain Therapeutic

The International Medical Device Regulators Forum (IMDRF) for software as a medical device (SaMD) consensus guidelines (28) state that a software intended to treat or prevent a serious disease would have to conduct well-controlled clinical trials to prove efficacy and seek pre-marketing authorization from a regulatory agency. CCT that is marketed for treating MCI or preventing AD would be viewed as a medical device and subject to pre-marketing regulatory oversight. CCT intended for use as a general wellness tool to improve mental speed would likely not be subject to such oversight. Recently, prescription digital therapeutics have been cleared by the US Food and Drug Administration (FDA) for use in substance abuse and sleep disorders, and apps for other diseases are in development (29).

We believe the most efficient regulatory path for CCT is to seek a marketing indication as a prescription digital therapeutic for the symptomatic treatment of MCI or very mild dementia. Such a path would be supported by the large public health threat posed by AD and the urgent need for scalable, low risk, cost-effective, home-based preventive treatments. The small to moderate effect sizes seen in MCI CCT trials to date are likely to be similar to those expected in ongoing anti-amyloid or anti-tau trials. Further, the safety of CCT is superior to most biologics/drugs being studied for MCI and the risk is minimal.

Recent FDA draft guidelines for acceptable outcomes in early AD trials of investigational drugs (30) provide a roadmap for CCT. The FDA guidance categorizes early AD into three stages—Stage 1 (pathological changes but no clinical deficits), Stage 2 (mild cognitive deficits but no measurable functional deficits), and Stage 3 (measurable cognitive and functional deficits). Stages 2 and 3 are analogous to early MCI and late-MCI. The FDA guidance suggests that in Stage 1 one or more biomarkers could serve as a primary basis for accelerated approval with the requirement for a post-approval confirmatory clinical study. In Stage 2, one or more neuropsychological tests (either effect on multiple tests or a large effect on a single test) could serve as the basis for approval. In Stage 3, a single integrated scale that measures both daily function and cognitive effects (e.g., Clinical Dementia Rating Scale) could serve as evidence of efficacy.

CCT manufacturers should seek advice from regulatory agencies and/or utilize the FDA's digital software pre-certification (Pre-Cert) program. In the US, given the lack of a predicate or product code, CCT for MCI would likely be viewed by the FDA as a Class III device (31); however, we believe that a *de-novo* application to request re-classification of CCT as a lower risk Class II device could be successful. If regulatory agencies view the existing studies of CCT in aging and MCI [such as those cited in (14–26)] as supportive, then only a single, methodologically rigorous, relatively short (e.g., 24-week) trial may be needed to gain such an indication. Alternatively a regulatory quality trial could also be conducted in the public interest through a public-private partnership involving one or more CCT companies or *via* a government grant. For example, our group is currently conducting an 18-month randomized trial of CCT vs. active control in carefully selected MCI patients with clinically meaningful cognitive (ADAS-Cog), functional (FAQ, UPSA), neuronal loss (hippocampal volume) and disease modifying (progression to dementia) outcomes (32).

Given the millions of elderly already doing CCT at home, it would also be insightful to analyze existing large registries to examine real world outcomes consistent with the FDA's total product lifecycle approach (31). Three areas of real world health analytics (RWHA) would be relevant for CCT—(1) patient reported outcomes such as daily activities; (2) user experience analytics such as engagement and compliance; (3) product performance (reliability, privacy, and cybersecurity). Updates on real world performance could be provided quarterly to public and regulators. Databases from large published RCTs (14–26) could be made available for such purpose with data sharing principles similar to the Dementias Platform UK or Alzheimer's Disease Neuroimaging Initiative (14, 33). The Human Cognition Project is one such CCT database that has already yielded useful insights and accessed by several academics (14, 34).

There are numerous CCT programs available on the market as wellness tools but none are currently cleared by regulators as a medical device and hence it is difficult for consumers and clinicians to choose among them. Regulatory clearance would increase trust and allow for greater scaling as a clinician supervised digital therapeutic (Figure 1). Future research to clarify the role of augmenting agents, such as off-label medications (e.g., vortioxetine), cholinesterase inhibitors, physical exercise, and other non-pharmacologic interventions, for CCT to achieve maximum efficacy as a cognitive enhancing strategy would also be useful. Future studies could also examine its utility in combination with anti-amyloid or anti-tau agents.

The COVID-19 pandemic has illustrated the demand for digital tools across the entire spectrum of healthcare. Ongoing studies are testing the utility of CCT for elderly subjects during the COVID pandemic (6, 31). For example, the TV-AssistDem a European multicenter randomized controlled trial evaluating a digital technology-based assistive integrated service to provide social connectedness and memory stimulation for MCI has rapidly adapted to the pandemic. Globally, reimbursement and regulatory burdens faced by digital tools before the pandemic have begun to diminish. The optimal features needed post-pandemic are difficult to predict at this time but hybrid models of home-based, tele-medicine and clinical based care will likely become the norm. Companies that integrate digital therapeutics with other modalities (e.g., digital diagnostics, digital pharmacy, live consults *via* tele-medicine) will best provide a seamless experience for consumers. Further, as our figure illustrates, features such as ease of use and ease of trouble shooting minimal supervision or ability to supervise by a caregiver, smooth integration with clinical medical record, remote access to results by doctors and therapists/psychologists for treatment monitoring, real time patient feedback, self-rated outcomes and real world analytics to track progress, and affordability would make it attractive to elderly in a post-pandemic situation. A patient-centered, real world health data sharing platform that can collect and aggregate siloed data sources across multiple health systems has recently been demonstrated (35). These lessons are highly relevant to optimize CCT as a clinical tool in MCI.

In summary, we believe that it is an important time for the field to advance CCT from a wellness product to a well-integrated, digital brain therapeutic platform *via* an appropriate regulatory pathway to help millions of elderly both during pandemics and in normal times.

## Author Contributions

KB and PD drafted the study. TG and DD provided critical edits. All authors helped with data interpretation.

## Conflict of Interest

PD has received advisory or board fees from several health and technology companies; PD owns shares in several companies and is a co-inventor on patents whose products are not discussed here. The remaining authors declare that the research was conducted in the absence of any commercial or financial relationships that could be construed as a potential conflict of interest.
